# Surgical Retrieval of a Broken Local Anesthetic Needle in the Pterygomandibular Space Using CBCT and C-Arm Guidance

**DOI:** 10.3390/diagnostics16060902

**Published:** 2026-03-18

**Authors:** Alexandru Nemțoi, Sorin Axinte, Ana Nemțoi, Vlad Covrig

**Affiliations:** 1 Faculty of Medicine and Biological Sciences, “Ștefan cel Mare” University of Suceava, 13 Universității Street, 720229 Suceava, Romania; alexandru.nemtoi@usm.ro (A.N.); vlad_cov@yahoo.com (V.C.); 2 Clinical Emergency Hospital “St. Joan the Newest”, 720224 Suceava, Romania; 3 Faculty of Dental Medicine, “Gr. T. Popa” University of Medicine and Pharmacy, 16 Universității Street, 700115 Iași, Romania; ana_bamboi@yahoo.com

**Keywords:** emergency treatment, dental anesthesia, oral surgical procedure, imaging, cone beam computed tomography

## Abstract

**Background and Clinical Significance:** Needle fracture during inferior alveolar nerve block is a rare complication, but it can nevertheless result in serious complications, especially when the fragment migrates into deep anatomical spaces like the pterygomandibular region. Accurate localization and safe retrieval are vital in preventing infection, chronic pain, neurovascular injury, and long-term functional impairment. **Case Presentation:** We present a case of a 27-year-old patient who had a fractured needle fragment from a local anesthetic procedure retained in the left pterygomandibular space. Cone beam computed tomography (CBCT) was carried out to verify the presence of the metallic foreign body and to define its exact three-dimensional position in relation to adjacent bone and soft tissue landmarks. The approach was transoral, and the surgery was done under general anesthesia. During the surgery C-arm fluoroscopy was used to help guide localization and retrieval, along with the help of radiopaque reference markers to assist in determining the trajectory. The fragment was removed without any issue. After the surgery, the patient’s condition improved well, and he showed no signs of functional deficits. **Conclusions:** The management of broken needle fragments in the pterygomandibular space can be safely and effectively done using a combination of preoperative CBCT and intraoperative C-arm guidance. This technique allows for exact location determination, minimizes unnecessary dissection of the tissue, and will make the surgery safer in complicated areas.

## 1. Introduction

The foundation of contemporary dentistry and oral maxillofacial surgery rests upon local anesthesia techniques, which allow a huge range of procedures to be safely and effectively performed. Stainless-steel needles have become disposable and metallurgy has advanced, so there are few needle-related complications. Local-anesthesia-needle fracture is nonetheless a rare but well-described complication. Most frequently it is associated with inferior alveolar nerve block anesthesia [1,2,3].

It has been estimated that the incidence of needle fracture during inferior alveolar nerve block (IANB) is extremely rare. The likely occurrence is approximately 1 in 14 million [4]. Even though it is a rare event, its clinical significance is noteworthy because it can lead to needle migration, local inflammation, infection, trismus, dysphagia and injury to vital neurovascular structures [2,5,6]. The inferior alveolar nerve and vessels and the lingual nerve, which anatomically communicate with deeper cervical space and parapharyngeal space, make the pterygomandibular space particularly vulnerable [7,8,9].

Several different factors have been implicated in needle fracture. These include sudden movement of the patient, application of excessive force while injecting, pre-bending of the needle, needle manufacturing defects, and inappropriate selection of needle gauge or length [2,3,10]. Needles with thinner diameters, especially 30-gauge needles, are prone to fracturing when inserted up to the hub or sharply angulated in dense soft tissue [3,11]. While several authors have suggested conservative management with radiographic monitoring in asymptomatic cases, most contemporary reports recommend prompt localization and removal due to the unpredictable risk of migration and medico-legal implications [5,8,12].

It is important to localize a fractured needle fragment accurately. Two-dimensional imaging modalities (panoramic or lateral radiographs), while useful in the initial evaluation, do not provide accurate three-dimensional spatial data [6,13]. Cone beam computed tomography (CBCT) has emerged as the imaging method of choice in the maxillofacial region, permitting the high-resolution three-dimensional assessment of the foreign body and its relationship to surrounding anatomical structures, whilst delivering relatively low radiation doses [1,6,14]. In some instances, it can be important to perform contrast-enhanced computed tomography in order to assess proximity to large vessels [9].

Obtaining a broken needle from the pterygomandibular space is a surgical challenge. A range of intraoperative localization techniques have been described, such as stereotactic methods, reference needles, metal detectors, navigational devices and endoscopy [7,15,16,17]. C-arm fluoroscopy is one of them. It offers real-time imaging, enabling dynamic intraoperative guidance for dissection while minimizing unnecessary trauma to tissues [9,16]. When used together with preoperative CBCT planning, C-arm-guided surgery is a valuable approach for safe and effective removal of fractured anesthetic needles from complex anatomical regions.

This article reports the retrieval of a broken local anesthetic needle in the pterygomandibular space. Cone beam computed tomography (CBCT) was done for diagnostic planning. During the surgery, C-arm fluoroscopy was used to localize the needle intra-operatively.

## 2. Case Report

### 2.1. Patient Information

A 27-year-old patient visited a private dental clinic for the extraction of the mandibular left first molar (3.6).

### 2.2. History and Presenting Complaint

Local anesthesia was started with inferior alveolar nerve anesthesia (at the level of the mandibular foramen (Spix spine)) as per the planned operating procedure. While performing needle anesthesia, the needle broke unexpectedly. The separate fragment was lost to sight and could not be retrieved intraorally. The left pterygomandibular region was where the fragment was suspected to be retained, according to the injection site and clinical condition. An inferior alveolar nerve block was performed using a disposable 25 mm 30-gauge needle (Transcodent, Schleswig-Holstein, Germany) attached to a standard aspirating dental syringe (Hu-Friedy, Chicago, IL, USA). The local anesthetic solution administered was 4% articaine with epinephrine 1:100,000 (Ubistesin Forte^®^, 3M ESPE, Seefeld, Germany). The needle fractured during injection at the level of the mandibular foramen.

On the day of the incident, the patient was referred for specialized assessment and went for evaluation at the Oral and Maxillofacial Surgery service of the ENT Department of Suceava County Emergency Clinical Hospital. The predominant issue presented was pain.

The patient presented to our department approximately 12 h after the incident. Surgical retrieval was performed within 18 h from the time of needle fracture.

### 2.3. Clinical Findings

The assessments were performed to determine any signs and symptoms that could suggest a case of possible retention and subsequent migration of a foreign body (FB), such as pain, restricted mouth opening, dysphagia, local inflammatory changes and neurosensory disturbances. The pterygomandibular space has an intricate anatomy. Moreover, it is closely related to the inferior alveolar neurovascular bundle and lingual nerve. Alongside this, the pterygomandibular space has deep fascial planes adjacent to it. Hence a surgical retrieval was decided upon, performed in the controlled environment of the operation theater.

### 2.4. Diagnostic Evaluation

Prior to surgery, cone beam computed tomography (CBCT) imaging was performed to confirm the presence of a fractured metallic fragment and to ascertain its precise three-dimensional location in the left pterygomandibular area. CBCT imaging provided accurate three-dimensional information about the relationship of the foreign body to the inner portion of the mandibular ramus, allowing for an optimized surgical plan and minimizing unnecessary dissection.

**Figure 1:**
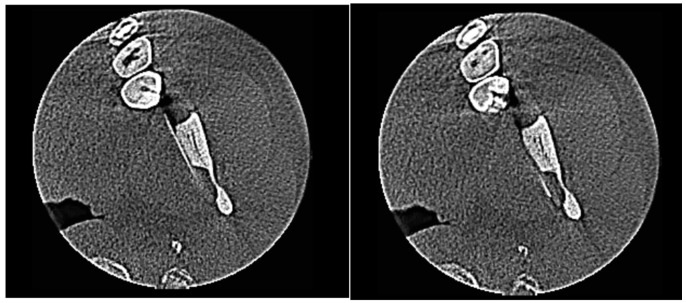
Axial CBCT slices confirming the retained needle fragment.

While viewing the axial CBCT slices, a linear hyperdense metallic structure compatible with a fractured needle fragment was observed deep in the soft tissue next to the mandibular ramus. The foreign body was clearly visualized on consecutive axial slices, establishing its retained status and ruling out unintentional superficial migration (Figure 1). Cone beam computed tomography imaging was performed using a KaVo OP 3D system (KaVo Dental GmbH, Biberach, Germany), with a field of view of 8 × 8 cm, voxel size of 0.2 mm, and exposure parameters of 95 kVp and 10 mA. The acquired DICOM datasets were analyzed using dedicated three-dimensional imaging software OnDemand3D™ (version 1.0, Cybermed Inc., Seoul, Republic of Korea) for multiplanar reconstruction and three-dimensional volume rendering.

**Figure 2:**
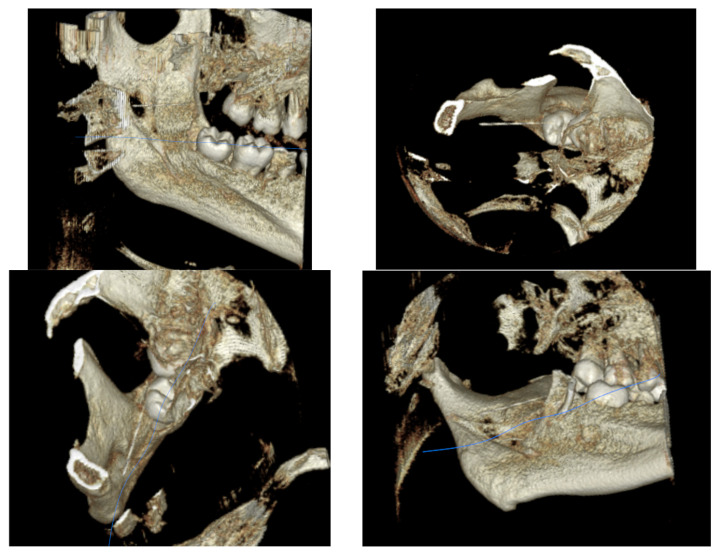
Three-dimensional CBCT reconstructions illustrating the spatial relationship of the foreign body. Blue line shows the dental needle.

We generated three-dimensional volume-rendered reconstructions to further enhance anatomical orientation and improve surgical planning. Through the available data and information, it was possible to reconstruct the trajectory of the foreign body in relation to the posterior mandibular region and the pterygomandibular space, as well as its depth of impaction. To obtain a better 3D understanding of the spatial relationship between the fragment and the mandibular ramus, multiple views were examined for an easier surgical roadmap towards a proposed transoral approach (Figure 2).

**Figure 3:**
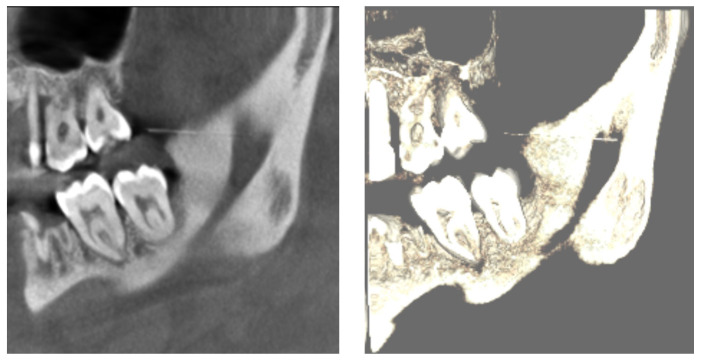
Multiplanar CBCT (panoramic) reconstructions highlighting depth and localization.

Furthermore, multiplanar reconstruction images (panoramic views) were inspected to confirm the location of the foreign body and also the depth concerning the cortical limits of the mandible. The images obtained aided in the decision to proceed with surgical retrieval under general anesthesia, as the fragment was not visible or palpable and was located in a narrow space. Image-guided intra operative localization was deemed necessary (Figure 3).

### 2.5. Surgical Procedure

**Figure 4:**
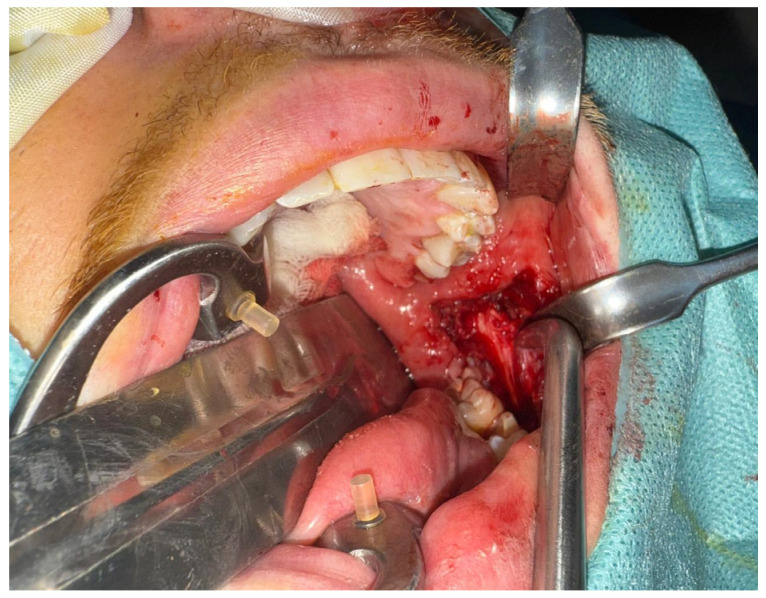
Intraoperative transoral exposure of the left pterygomandibular region.

**Figure 5:**
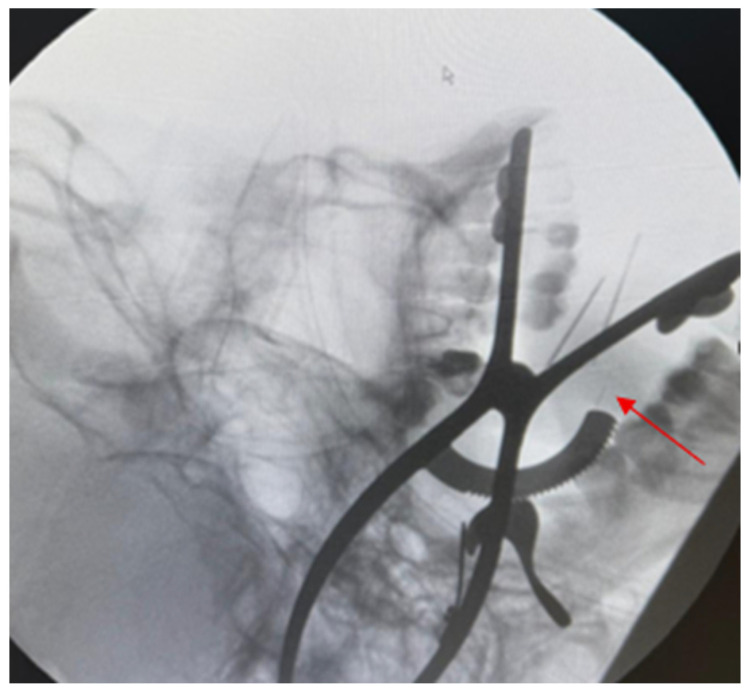
Intraoperative C-arm fluoroscopy for foreign body localization. Red arrow shows the dental needle.

The foreign body was deep, and most likely removal would be difficult, so we planned to do it under general anesthesia to aim for patient immobility, airway protection, and also safe exploration. For direct access to the medial aspect of the mandibular ramus and to minimize external scarring, a transoral approach was chosen. An incision was made at the front of the mandibular ramp after sufficient exposure was achieved. Careful dissection of the medial surface of the left ramus was done towards the pterygomandibular space with good hemostasis, taking constant care not to damage adjacent structures (Figure 4). Intraoperative imaging guidance was deemed necessary due to the needle fragment being located at a depth that could not be identified by either visualization or palpation. As a result, a C-arm fluoroscopy system was employed for real-time radiographic feedback to improve localization accuracy (Figure 5). The small size of the fragment and the narrow surgical corridor limited spatial orientation during exploration. To address these limitations, two intravenous access trocars were introduced as radiopaque reference markers. The landmarks helped the surgical team more accurately estimate the three-dimensional relationship between the dissection plane and the foreign body under fluoroscopy. To optimize the surgical trajectory, targeted dissection towards the fragment was realized whilst minimizing dissection of the non-targeted region; sequential fluoroscopic views were taken.

**Figure 6:**
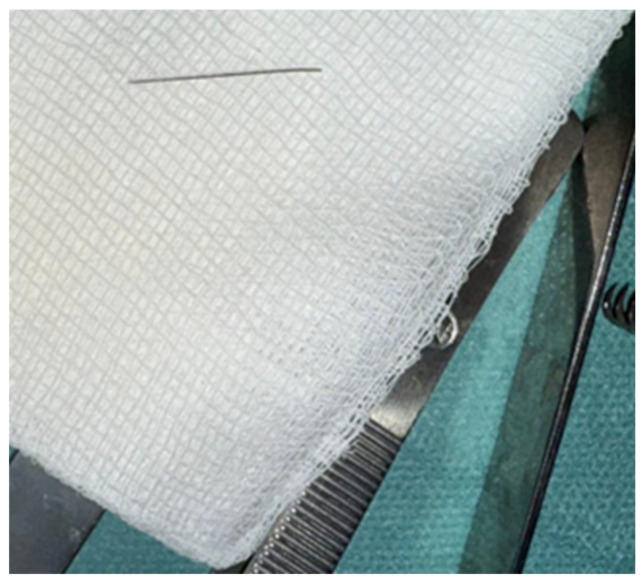
Retrieved foreign body (fractured needle fragment).

Through a gradual and controlled exploration, along with the assistance of C-arm imaging and radiopaque markers, we were able to confirm that the fractured needle segment was located within the left pterygomandibular space. The foreign body was held and removed successfully (Figure 6). The site of the surgery was examined for any remaining pieces and for damage to surrounding tissue. When complete retrieval was confirmed, the operative field was irrigated, hemostasis secured and the wound was closed in layers with resorbable sutures.

Intraoperative fluoroscopic guidance was achieved using a mobile C-arm system (Ziehm Solo FD, Ziehm Imaging GmbH, Nuremberg, Germany). Radiopaque reference markers consisted of sterile intravenous access trocars (BD Venflon™ Pro Safety, Becton Dickinson, Helsingborg, Sweden). General anesthesia was induced using a standard balanced anesthesia protocol with endotracheal intubation, according to the institutional anesthesia guidelines.

### 2.6. Outcome and Follow-Up

Following surgery, the patient’s subsequent observation revealed favorable clinical progress. Clinical follow-up was performed for one month after surgery, and during this period, the patient showed progressive recovery without signs of infection, trismus, or functional impairment.

### 2.7. Clinical Relevance

This case shows the importance of timely referral and image-guided retrieval of deeply embedded foreign bodies in the maxillofacial region, where complex anatomy may increase surgical risk.

## 3. Discussion

### 3.1. General Considerations

Despite the rarity of needle fracture when using dental local anesthesia, this complication is clinically significant as the needle can become embedded in deep soft tissue, be difficult to retrieve, and migrate [18]. In their study, Kim et al. stated that even though inferior alveolar nerve block is a routine procedure and generally safe, needle breakage continues to be reported sporadically and can cause major complications when the fragment disappears into deep anatomical spaces [11]. Likewise, Malkawi et al. pointed out that even rare complications, by virtue of the anatomy and the distance to neurovascular structures, may have serious consequences in the head and neck region [9].

### 3.2. Etiology and Predisposing Factors

Several factors are associated with the fracture of a needle during mandibular nerve block. Sudden movement of the patient at the time of injection has often been described as a major precipitating factor, producing mechanical stress at the weakest point of the needle [6,11,19,20]. Moreover, Villalobos et al. further described additional contributory factors, including technical mistakes, the use of pre-bent needles, and possible manufacturing defects [6]. According to Erdil et al., 30-gauge and thinner needles may be more prone to bending and fracture when force is applied and the needle direction is changed with a deeply inscribed needle [1].

In the present case, the fracture happened during an inferior alveolar nerve block at the level of the mandibular foramen, and the fragment immediately became non-visible. This mechanism coincides with earlier cases [1,6,9,11].

### 3.3. Clinical Presentation and Rationale for Management

The retained needle’s clinical significance depends on its location and depth of penetration as well the presentation of any symptoms. Villalobos et al. described cases in which retained fragments remained asymptomatic and were managed conservatively with imaging [6]. Malkawi et al. stated that it would be better to remove them early to prevent pain, trismus, infection and injury of adjacent structures if a fragment is in the pterygomandibular space [9].

Our patient experienced pain on arrival and imaging suggested a deep position in the pterygomandibular region. That area has complex anatomical features, and it also carries the risk of further migration; thus, surgical retrieval was considered the best therapeutic option, as per the recommendation of various authors [1,6,9,11].

### 3.4. Remove vs. Observe: How Our Approach Fits in the Spectrum

One controversy in the literature is whether an asymptomatic retained needle fragment should be routinely removed. Villalobos et al. reported a situation where the fractured fragment migrated in the first weeks to months following the fracture but then stabilized, and after reassessing risk and the fact that the patient had no real symptoms, they decided on a radiographic follow-up as opposed to repairing it surgically [6].

In contrast, other authors defend early removal whenever possible because of the risk of migration towards more delicate anatomical areas, the risk of infectious/inflammatory complications, and the psychological burden of retention of a sharp metallic foreign body [9,11].

In this case, a fragment was found deep inside the patient who was experiencing pain. In addition, the proximity of the pterygomandibular region to the inferior alveolar neurovascular bundle, the lingual nerve and adjacent deep fascial planes made a conservative ‘watchful’ strategy less appropriate. This in line with the literature that indicates that the presence of these symptoms (pain, trismus, dysphagia, inflammatory signs) and an unfavorable anatomical location strengthen the indication for early surgical retrieval [6,9].

### 3.5. Importance of Imaging and Localization

Precise localization is crucial for safe and effective retrieval. Villalobos et al. demonstrated that although the presence of metallic foreign bodies can be diagnosed using panoramic radiography, advanced imaging techniques, especially the use of computed tomography, offer better spatial resolution and understanding of the relationship with adjacent structures [6]. According to Kim et al. [11], three-dimensional imaging is helpful to corroborate the location of the fragments and to assist in surgical planning. According to Malkawi et al., CBCT is the first imaging choice for s lesions, though with further cross-checking imaging if deeper (radicular) migration to vital structures is suspected [9].

In the present case, preoperative CBCT was used to confirm the retained fragment and assist in surgical planning, consistent with the imaging strategies suggested in the literature [6,9,11].

### 3.6. Surgical Approach and Anesthesia Considerations

This method was chosen since it gives access to the ramus’ medial aspect through the mouth without any external scarring. Kim et al. reported an efficient retrieval through an intraoral incision and dissection along the ramus using a principle of maintaining anatomical orientation [11]. Malkawi et al. used a transoral approach and remarked on the technical difficulty associated with restricted visual access within a narrow corridor [9].

Researchers believe unilateral or even bilateral retrieval under local anesthesia may be possible in selected cases. Despite this, they believe general anesthesia is preferable in view of deeper fragments lying in high-risk anatomical regions to avoid movement and allow for safer exploration [1]. In our case, we put the patient under general anesthesia to ensure maximum safety, airway protection, and controlled dissection within the pterygomandibular space.

### 3.7. Role of Intraoperative Guidance and Reference Markers

Retrieving fractured needles can be difficult because needle fragments are small and it can be difficult to precisely orient the dissection in three dimensions. According to Malkawi et al., C-arm internal fluoroscopy helps in the orientation and retrieval of deep anatomies [9]. According to Kim et al., a metallic reference marker helps in intraoperative orientation according to the imaging findings.

In this case real-time C-arm fluoroscopy was aided by inserting two intravenous access trocars as radiopaque reference markers. Through enhanced spatial orientation and triangulation of the fragment location, further dissection and handling of surrounding tissue could be avoided. In its essence, this approach is similar to the marker-assisted and image-guided methods reported in [9,11,12,21,22,23].

### 3.8. Postoperative Outcome and Clinical Implications

Earlier studies have shown good success of the removal of fractured needle segments using well-planned image-guided augmented strategies. Both Kim and Malkawi reported uneventful healing after controlled transoral retrieval aided by sophisticated imaging [9,11]. The postoperative course in our patient was also favorable with no early complication.

Overall, this case supports the concept that deeply located needle fragments within the pterygomandibular space can be safely and effectively removed when a structured diagnostic approach, such as CBCT, and intraoperative guidance with fluoroscopy and radiopaque reference markers are employed.

## 4. Conclusions

The fracture of a needle during inferior alveolar nerve block is rare, but has clinical significance. It becomes clinically significant when the fragment has become non-visible and retained within deep anatomic spaces, such as the pterygomandibular region. This case of a 27-year-old patient who presented with pain after needle fracture during mandibular anesthesia emphasizes timely referral to specialist and risk assessments, as this space is in proximity to major neurovascular structures.

The use of preoperative CBCT aided in confirming the retained fragment and identifying its location to facilitate proper planning for surgery. Retrieval under general anesthesia via a transoral approach permitted controlled access to the medial aspect of the mandibular ramus without external scarring. The localization of the intraoperative fluoroscopy C-arm improved significantly. Furthermore, the use of two intravenous access trocars as radiopaque reference markers greatly aided spatial orientation and helped to dissect towards the foreign body.

The needle part was removed without incident and postoperative recovery was unremarkable. Overall, it can be concluded from this report of our experience that preoperative 3D imaging in addition to intra-operative fluoroscopy and the use of radiopaque landmarks is valuable for the successful and safe retrieval of deep needle fragments in anatomically complex areas.

## Data Availability

Data are available upon request.
